# Highlight: Unmasking the Supergene Beneath the Ruff's Mating Strategies

**DOI:** 10.1093/molbev/msad249

**Published:** 2023-12-07

**Authors:** Casey McGrath

In the colorful world of avian courtship, the ruff (*Calidris pugnax*) is in a league of its own. Breeding in marshes and wet meadows across Eurasia, the males of this medium-sized sandpiper species are well-known for their distinctive mating strategies, which range from flamboyant territorial displays to cunning mimicry. These behaviors, along with striking differences in plumage, are determined by a single genetic region known as a supergene. Supergenes are clusters of genes that control complex traits. They are often associated with a chromosomal inversion, in which the gene order is reversed along the chromosome compared with the wild-type allele; this serves to suppress recombination, allowing a set of traits to be coinherited. While there are potential benefits to preserving favorable combinations of genetic variants, this lack of recombination can also lead to the accumulation of deleterious mutations within the supergene over time. However, a new study published in *Molecular Biology and Evolution* (Hill et al. 2023) has revealed a remarkable evolutionary paradox, as the supergene that underlies male mating strategy in the ruff exhibits a surprisingly low mutation load (Hill et al. 2023). The study's findings therefore challenge our understanding of the evolution and persistence of supergenes in nature.

**Fig. 1.**
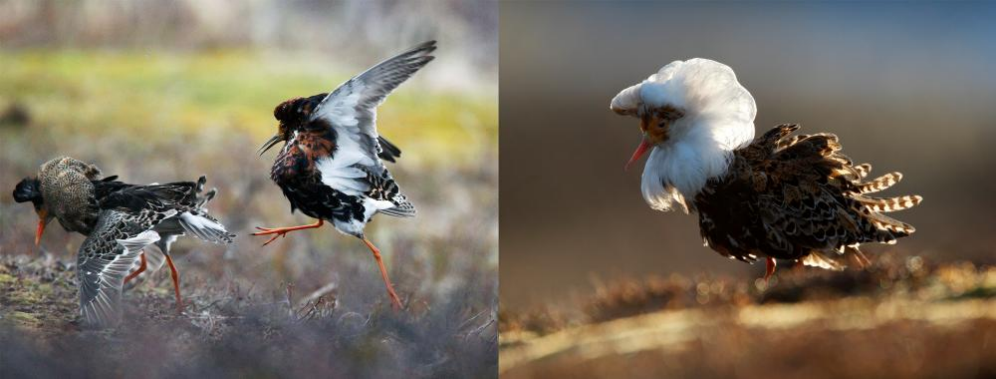
Male phenotypes in the ruff. (Left) Independent ruff males interacting at a lek. (Right) A Satellite ruff male with pale ornamental feathers at a lek. Photos courtesy of Tom Schandy.

Ruff males have long captured the attention of scientists and birdwatchers alike due to their showy mating displays and outlandish plumage, resembling the extravagant collars worn in the sixteenth century that inspired the species’ name. There are actually three distinct types of male ruffs, known as Independents, Satellites, and Faeders, which differ in behavior, plumage, and body size (Fig. 1). “Independents have spectacular ornamental feathers, and these males defend territory on the lek [mating grounds],” says Leif Andersson, the lead author of the new study. “Satellites have light-colored ornamental feathers and do not defend territory on the lek but allow Independent males to dominate them. This behavior helps Independent males attract females that are ready to mate; the advantage for the Satellites is that they get access to the mating ground without the need to spend energy defending territory on the lek. Faeders are nonterritorial, female-mimics with no ornamental feathers. They sneak around on the lek and try to mate with females.”

Interestingly, the Satellite and Faeder phenotypes are determined by the presence of an inversion that harbors about 100 genes. “The Faeder haplotype is an intact inversion while the Satellite haplotype originated after genetic recombination between the Independent and Faeder haplotypes,” continues Andersson. In addition to carrying one of the inverted haplotypes, all Satellite and Faeder males carry one Independent haplotype, as the presence of two copies of the inversion (in the recessive or homozygous state) is lethal.

The ruff supergene has long puzzled Andersson and his research team. “When we first discovered the ruff supergene,” says Andersson, “we were amazed that the sequence divergence between the inversion alleles and the wild-type allele was as high as 1.4%. This is higher than the sequence divergence between humans and chimpanzees and suggested a split about 4 million years ago based on the estimated substitution rate for birds. The inversion alleles are recessive lethal, most likely because the inversion breaks an essential gene. Thus, the question that emerged is how can a recessive lethal be maintained for 4 million years?”

To investigate this mystery, the researchers employed cutting-edge genomic sequencing techniques to create highly contiguous genome assemblies for both the Independent and Satellite haplotypes. They used these assemblies alongside previously published whole-genome data to assess the mutational load of the inverted supergene. As noted by Andersson, “Population genetic theory predicts that supergenes should accumulate genetic load [e.g. deleterious mutations] due to relaxed purifying selection, in particular if the supergene is a recessive lethal like the ruff supergene is.”

Surprisingly, however, the researchers found no substantial accumulation of repetitive elements and only a modest mutation load on the Satellite and Faeder haplotypes. This unexpected finding forced the study's authors to reassess their assumptions about the ruff supergene. “I really had to reevaluate the way that I thought about supergenes as we continued to find evidence of recent purifying selection where there should not have been any,” notes Andersson.

The authors propose two potential scenarios to resolve this paradox. First, the inversion may have only recently acquired its recessive lethality. If an older version of the supergene was more common and not a recessive lethal, recombination could occur in ruffs carrying two copies of the inversion, allowing deleterious mutations to be removed through purifying selection. An alternative hypothesis, which is favored by the authors, is that the supergene was introduced by introgression from another species or subspecies. In this scenario, hybridization between a ruff and another species led to introduction of the supergene into the ruff genome, and its persistence was then favored by selection because it kept together alleles contributing to a successful male mating strategy. While the study authors were unable to identify the lineage that may have contributed the inversion, they note that given the estimated timeline, the donor species may now be extinct.

This study highlights the complex forces governing male mating strategies in the ruff and supergenes in general. “Inversions are easy to find with modern genomic tools but are difficult to understand,” notes Andersson. “However, it should be very interesting to analyze gene expression in multiple tissues from the different morphs and try to understand which of the genes in the inversion contribute to the spectacular differences between morphs.” While their genomic data have so far unearthed two potential candidate genes—one involved in testosterone metabolism and one that may influence ornamental feather coloration—additional transcriptomic data are needed to answer this question. Unfortunately, such data may be difficult to obtain: “The major challenge with this suggested gene expression study,” says Andersson, “is that this is a wild species, and it is not easy to put together the large collection of samples that will be needed for a comprehensive analysis.” Despite this hurdle, further research into this remarkable model system promises to provide a deeper understanding of the origin, persistence, and evolutionary trajectories of supergenes.
